# Feasibility and Effectiveness of a Social Network-Based Intervention for Adolescents Undergoing Weight Loss Treatment: A Randomized Controlled Trial

**DOI:** 10.3390/nu17162586

**Published:** 2025-08-08

**Authors:** Sofia Marques Ramalho, Pedro F. Saint-Maurice, Diana Silva, Helena Ferreira Mansilha, Eva Conceição

**Affiliations:** 1 Psychology for Development Research Center (CIPD), Institute of Psychology and Education Sciences, Lusíada University, Rua de Moçambique 21 e 71 (Aldoar), 4100-348 Porto, Portugal; 2 Champalimaud Foundation, 1400-038 Lisbon, Portugal; 3 Unidade Local de Saúde de São João, E.P.E., 4200-319 Porto, Portugal; 4 Faculdade de Ciencias da Nutrição e Alimentação, Universidade do Porto (FCNAUP), Rua do Campo Alegre nº 823, 4150-180 Porto, Portugal; 5 Centro Hospitalar e Universitário de Santo António, Centro Materno Infantil do Norte (CMIN), Largo da Maternidade de Júlio Dinis, 4050-651 Porto, Portugal; 6 Faculty of Psychology and Education Sciences, Center for Psychology at University of Porto, University of Porto, 4200-135 Porto, Portugal

**Keywords:** web-based intervention, social networks, Facebook, pediatric obesity, adolescent, health promotion, eating behaviors

## Abstract

Background/Objectives: Digital interventions can have a positive effect on the health-related behaviors of adolescents. However, it is unclear if social network-based interventions using Facebook can help to optimize medical treatment as usual (TAU) for adolescent obesity in public health care centers. We examined the feasibility, usability, and effectiveness of APOLO-Teens, a Cognitive Behavioral Therapy Facebook-based intervention as a supplement to TAU on changing eating habits/behaviors, physical activity levels, and psychological functioning of adolescents with overweight/obesity. Methods: This was a Randomized Controlled Trial (Trial registration number: NCT04642222). One-hundred and thirty-five adolescents aged 13 to 18 years (67.5% females) were randomly assigned to the TAU control group (*n* = 66) and the APOLO-Teens intervention group (*n* = 69). Intervention outcomes were measured at baseline and the end of the intervention (6 months later). Using per-protocol analysis, the sample size retained for final analysis included 77 participants (Control group = 39; Intervention group = 38). Two-way mixed ANOVAs were used to test within-and between-group changes. Results: The APOLO-Teens social network-based intervention was feasible (adherence rate: 85.5%) and the intervention group had a significant increase in fruit consumption (F (1,35) = 6.99, *p* = 0.012; significant group-by-time interaction). Both groups increased vegetables on the plate consumption and decreased pastries/cakes intake, depressive symptomatology, grazing eating pattern, and BMI z-score (*p* < 0.05; significant time interaction). Conclusions: The APOLO-Teens social network-based intervention was feasible, and the effectiveness results suggest that it can be a beneficial supplementary intervention to TAU in adolescent obesity.

## 1. Introduction

Obesity in adolescence is related to short- and long-term physical and psychological consequences and is also a risk factor for obesity in adulthood [1,2]. Some of the consequences of having excessive weight in adolescence include metabolic and cardiovascular disorders (e.g., development of metabolic syndrome), poor academic performance, depression, increased risk for developing disordered eating behaviors, and lower quality of life [3]. Although genetic factors are related to the individual predisposition for weight gain, environmental, behavioral, and family factors can also play a significant role. Some of the behaviors that can increase the risk for obesity include the intake of high caloric foods and sweetened beverages, a reduction in fruit and vegetable consumption, and an increase in the time spent on sedentary activities [3,4,5].

Although global overweight prevalence during childhood and adolescence is projected to stabilize from 2022 to 2050, obesity rates are expected to rise markedly between 2022 and 2030, and this upward trend is expected to persist through 2050. Timely public health measures are required to mitigate this growing public health challenge [6]. Multicomponent interventions combining diet, physical activity, and behavioral therapy are the first-line treatment for adolescent overweight/obesity [3,7]. They can produce positive changes in health behaviors and appear to be more effective than single-component interventions at enhancing the health-related quality of life and at reducing body weight in adolescence [8,9,10,11].

However, these interventions are rarely offered in public health care settings, such as hospitals, due to a lack of human/economic resources, evidence-based knowledge, and counseling skills [12]. Given the rising rates of overweight and obesity among adolescents, particularly, in low-income families [1,6,13] and adolescents’ lack of motivation for lifestyles changes, alternatives are needed so that multicomponent interventions for adolescent obesity can be more easily implemented in public health care services to achieve mass dissemination [14].

Evidence suggests that social networking sites can have a positive effect on health behavior change [15,16,17]. Previous studies using Facebook^®^ to deliver weight-loss interventions showed high feasibility and acceptability [18,19,20,21,22]. In particular, social media networks are widely used by adolescents and have the potential to reduce the high attrition rates of hospital standard treatment for overweight/obesity [22,23,24]. For example, Parks and colleagues conducted a pilot trial that showed that private Facebook groups were a feasible adjunct to improve adherence to medical weight management in youth with severe obesity [24]. However, results regarding weight loss are mixed. Some studies using Facebook-based interventions showed significant Body Mass Index (BMI) reduction in college students [25]. On the other hand, a three-arm randomized controlled trial testing a 12-week Facebook lifestyle counseling intervention with and without physical activity monitorization to adolescents with overweight/obesity in school settings showed no significant intervention effects on physical activity levels and BMI [20].

Despite promising results, the evidence on social media-based interventions in pediatric obesity remains limited. Existing studies vary widely in design, population (school vs. clinical settings), and intervention components, making it difficult to draw clear conclusions regarding their effectiveness [19,20,26,27]. Moreover, no previous studies explore the use of a Facebook-based intervention to provide Cognitive-Behavioral Therapy (CBT) for adolescents undergoing weight loss treatment in public hospital settings.

Therefore, this study addresses these gaps by evaluating the feasibility, usability, and effectiveness of APOLO-Teens, a manualized CBT intervention delivered via Facebook^®^, and a self-monitoring web application with personalized feedback, as an adjunct to standard medical/nutritional treatment as usual (TAU) [28] in a public hospital setting.

APOLO-Teens targets obesity-related behaviors and aims to promote the adoption of healthy eating habits and lifestyle behaviors. The APOLO-Teens was designed to be part of a multidisciplinary intervention for obesity that includes the medical/nutritional treatment as usual (TAU). To our knowledge, this is the first randomized controlled trial to test a manualized Cognitive Behavioral Therapy (CBT) Facebook-based intervention for adolescents with overweight and obesity undergoing medical/nutritional treatment at a public hospital.

Thus, the purpose of the present study was to test the feasibility, usability, and effectiveness of the Facebook-based intervention APOLO-Teens as a supplementary intervention to optimize TAU for adolescents with overweight and obesity undergoing hospital weight loss treatment. We hypothesized that, compared to TAU alone, the APOLO-Teens intervention would lead to greater improvements in health-related behaviors (e.g., fruit intake, physical activity).

## 2. Materials and Methods

### 2.1. Study Design

This is a randomized control trial with two groups: the TAU control group (TAU group) and the APOLO-Teens intervention group (APOLO-Teens group). Participants were recruited in their medical appointment in two public hospitals in the north of Portugal from October 2015 to December 2017. This study had approval from the two hospital ethical committees and the University Ethics Commission. It was registered on the ClinicalTrials.gov registry (NCT04642222).

Participants accepting participation signed an informed consent form and responded to a set of questionnaires. After completing the baseline assessment, participants were consecutively randomized using the Research Randomizer web-based program (http://www.randomizer.org/, accessed on 1 October 2015) into the APOLO-Teens group or the TAU group by a researcher not involved in data management. The allocation ratio was 1:1. Randomization was stratified by sex to keep a balanced distribution of boys and girls across groups. This study comprised online assessments at baseline (Tb) and end of intervention (Tf) (or the corresponding period for the control group). Anthropometric data was collected from the hospital clinical charts at baseline, three months after the beginning of the intervention, and at the end of the intervention. Participants in the APOLO-Teens group received the APOLO-Teens web-based intervention delivered by Facebook^®^ in addition to their treatment as usual (TAU). APOLO-Teens intervention facilitator and participants were not blinded to the treatment group. At the end of the study, participants randomized to the control group were offered access to the intervention materials.

### 2.2. Participants and Recruitment

**Figure 1:**
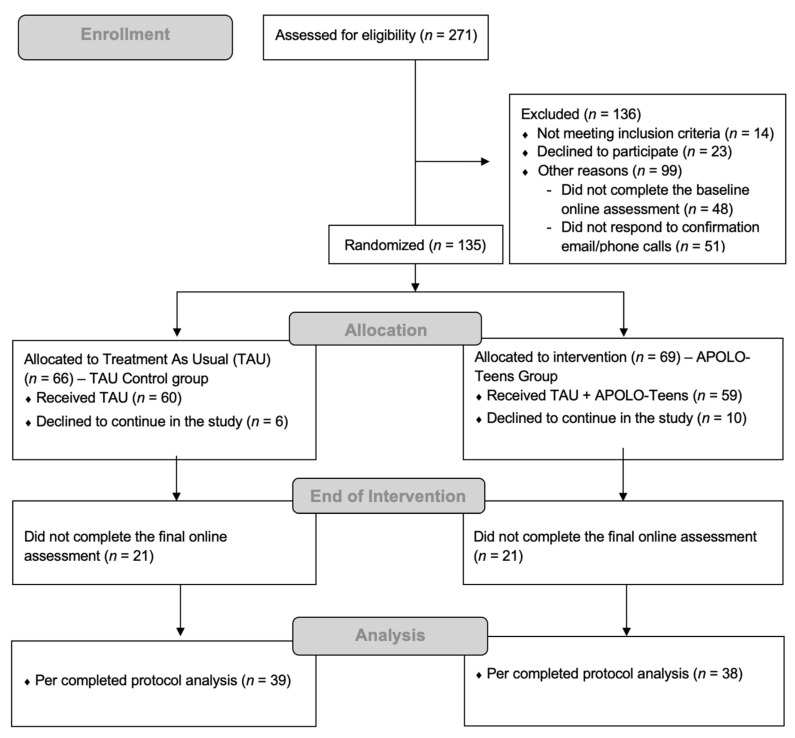
APOLO-Teens randomized controlled trial flow diagram.

Participants were adolescents, aged between 13 and 18 years old, with overweight or obesity (BMI z-score ≥ 1 (World Health Organization) under the TAU weight loss. Adolescents were recruited directly by a psychologist at the public hospitals in the context of a previously scheduled appointment with a nutritionist/pediatrician (TAU) after parental and adolescent written consent. Inclusion criteria included having a Facebook^®^ account and access to the Internet at least three times per week. Exclusion criteria included (1) medical conditions that affect weight; (2) specific learning difficulties that prevented adolescents from reading and understanding written text; (3) ambulatory movement limitations; (4) and BMI z-score above 4 or an indication for bariatric surgery (please see Figure 1).

### 2.3. Measures

Sociodemographic Questionnaire: At baseline (Tb) participants were asked about age, sex, educational level, and parents’ demographic data.

#### 2.3.1. Primary Outcomes:

##### Feasibility and Usability

Feasibility: The adherence rate (the proportion of adolescents invited who participated in the intervention) and the attrition rate (proportion of adolescents who dropped out during the intervention) were estimated to evaluate the feasibility of the APOLO-Teens web-based intervention.

APOLO-Teens Usability Questionnaire: This is a 30-item questionnaire (designed by the research team) applied 3 months after the beginning of the intervention to the APOLO-Teens group. It uses a 5-point rating scale (from 0 = “nothing” to 4 = “extremely”) to evaluate the usability perception of the intervention group participants regarding the following APOLO-Teens web-based intervention domains: weekly videos/tasks, chat sessions, self-monitoring system, and overall utility/satisfaction (Please see Table 1). The higher the total score, the higher the usability.

##### Behavioral

Foods/beverages frequency questionnaire: This questionnaire assesses the frequency of consumption of soup (1 plate), fruit (1 piece), vegetables (1/4 of the plate), sweetened beverages drinks (1 glass) and pastries/sweets (1 piece) in the previous week (0 = no intake; 1 = once a week; 2 = 2 to 4 times per week; 3 = 5 to 6 times per week; 4 = once a day; 5 = twice a day; 6 = three times a day; 7 = 4 times a day; 8 = 5 times a day; 9 = 6 or more times per day). Higher values indicate higher consumption.

Youth Activity Profile (YAP) [29]: This is a 15-item questionnaire to evaluate physical activity and sedentary behaviors in youth on the previous seven days through a 1–5 Likert scale. It generates minutes of moderate to vigorous physical activity per week at school, out of school, and also minutes per week spent on sedentary behaviors.

#### 2.3.2. Secondary Outcomes

##### Problematic Eating Behavior

Children’s Eating Attitudes Test (ChEAT) [30,31]: It evaluates eating behaviors disturbance through a 26-item scale. ChEAT comprises four subscales: (1) fear of getting fat, (2) restrictive and purging behaviors, (3) food preoccupation, and (4) social pressure to eat. Higher scores indicate more eating disturbance (McDonald’s for this sample: ChEAT total score = 0.87).

Repetitive Eating Questionnaire (Rep(eat)-Q) [32]: This is 12-item self-report measure to measure grazing-type eating patterns by a Likert 7-point scale. Higher scores indicate the existence of a grazing-type eating pattern (McDonald’s for this sample = 0.83).

##### Psychological Functioning

Depression Anxiety Stress Scales (DASS-21) [33,34]: This is a 21-item instrument that generates three subscales assessing depression, anxiety, and stress. For the present study, just the depression subscale will be used, with higher scores indicating more depressive symptomology (McDonald’s for this sample = 0.85).

Pediatric Quality of Life Inventory-PedsQL [35]: It is a 21-item measure assessing health-related quality of life in children and adolescents. PedsQL includes four subscales: physical functioning, emotional functioning, social functioning, and school functioning as a total score. Higher scores indicate higher health-related quality of life (McDonald’s for this sample: total score = 0.88).

##### BMI z-Score

BMI z-score was calculated based on weight and height registered in the hospital clinical charts from the TAU medical appointments during the intervention period (or equivalent time for the TAU control group). Weight was measured by a digital balance (Tanita^®^ model TBF-300) and height by a portable stadiometer (Seca^®^ model 206). BMI z-scores for age and sex were calculated by WHO Anthroplus software 3.2.2. version. Assessments conducted within 30 days of baseline, middle (3 months), and end of intervention assessments were used.

### 2.4. Intervention

#### 2.4.1. Treatment As Usual (TAU)

TAU is the standard intervention offered in Portuguese public hospitals accessible at a low cost for the overall population. For this study, the TAU comprised three nutritional appointments (at baseline, 3 and 6 months after baseline). TAU 30 min appointments included a physical examination (weight, height) and personalized dietary/lifestyle recommendations regarding the frequency of healthy/unhealthy food consumption, reduction in screen time, and the increase in daily moderate to vigorous physical activity. TAU did not integrate any structured lifestyle program or psychological intervention tailored to weight loss. The TAU intervention is common to the experimental and control groups. No statistically significant differences were found between the proportion of TAU nutritional appointments between the intervention and control groups (APOLO-Teens group M = 1.6, SD = 0.7; TAU Group M = 1.9, SD = 0.8; Z = 1.42, *p* = 0.16).

#### 2.4.2. APOLO-Teens Web-Based Intervention

The APOLO-Teens was designed to optimize TAU by promoting the adoption of healthy eating habits and lifestyle behaviors. Particularly, it aims to promote higher consumption of fruits and vegetables, increase physical activity levels, reduce sedentary time, enhance psychological well-being, and facilitate weight loss.

The APOLO-Teens web-based intervention comprises three main components: (1) a manualized intervention implemented via Facebook^®^ in private groups (of 10–12 participants). The intervention includes psychoeducational weekly videos with cognitive–behavioral therapy strategies and daily psychoeducational/motivational images on 6 different monthly topics. Each topic was discussed with a set of weekly cognitive–behavioral tasks (Online Resource—Figure S1); (2) a weekly self-monitoring system (the APOLO-Teens web application) with automatic feedback messages assessing the following behaviors: hours of physical activity, sedentary time, and consumption of fruits and vegetable (Online Resource—Figure S2); and (3) monthly chat sessions (20–30 min) via Facebook Messenger^®^ coordinated by an MSc Psychologist and available on request from the participants. This intervention was fully implemented by a psychologist with a Master’s degree trained in cognitive–behavioral therapy. A detailed description of the intervention has been published [28].

### 2.5. Statistical Analyses

Descriptive statistics were performed to describe participants’ sociodemographic and anthropometric baseline characteristics. Validated self-report instruments were used to assess depressive symptoms and eating behaviors; however, no clinical cutoff scores were applied, as the analyses focused on continuous data to reflect the full spectrum of symptom severity. Chi-square tests (χ2), *t*-test for independent samples, and Mann–Whitney U tests were used, according to variables distributions, to test differences between the TAU group and APOLO-Teens group at baseline (Tb). We conducted logarithmic and square root transformations to our measures if the assumption of normality was violated.

Two-way mixed ANOVAs (with main effects for condition/time and interaction effects) were performed to assess differences in the patterns of change in primary and secondary outcome measures between baseline and end of intervention assessments. Partial Etas (η_p_^2^) were reported as an estimation of the effect size (small effect = 0.01; medium effect = 0.06; and large effect = 0.14) [36].

The minimum sample size to detect a medium effect size of 0.25 (Cohen’s d) on primary outcomes considering the following assumptions: error of 5%, power of 90%, significant at alpha = 0.05, and accounting for 20% attrition was 55 adolescents (G*Power 3.1).

The goal of this preliminary study was to assess efficacy under conditions of adequate engagement; thus all analyses were conducted using a per-protocol analysis, excluding adolescents who were randomized but did not enroll in all the assessment moments. The IBM^®^ SPSS^®^ Statistics 22.0 (SPSS Inc., Chicago, IL, USA) was used for data analyses. *p* values < 0.05 were considered significant.

## 3. Results

### 3.1. Participants

Figure 1 presents the flow chart of participants throughout the study. Of the 135 participants that accepted to participate in the study, 69 (51.11%) were randomized to the APOLO-Teens group and 66 (48.89%) to the TAU group. The 69 participants allocated to the APOLO-Teens group were distributed by six Facebook^®^ private groups of about 12 participants each. A total of 42 participants (21 from the TAU group and 21 from the APOLO-Teens group) did not respond to the end of the intervention online assessment and were excluded from the final analysis. The final analytical sample included 39 participants in the TAU group and 38 participants in the APOLO-Teens group.

Participants’ demographic and anthropometric baseline characteristics are shown in Table 2 for the TAU group and the APOLO-Teens group. No differences between the two groups were found regarding sex (χ2 (1) = 1.30, *p* = 0.255) or age (U = 718.50, *p* = 0.814). Baseline differences between the TAU group and the APOLO-Teens group were explored for primary and secondary outcome measures. No differences were found, except for health-related quality of life (PedsQL) (U = 530.50, *p* = 0.046), which was significantly higher for TAU group at baseline (M = 79.78, SD = 15.67 vs. M=74.43, SD = 15.05).

### 3.2. Feasibility and Usability of APOLO-Teens Web-Based Intervention

Out of the 69 participants initially included in the APOLO-Teens group, 59 stayed as members in the Facebook^®^ private group until the end of the intervention suggesting that the APOLO-Teens intervention had a high adherence rate (85.51%) and an attrition rate of 14.49%.

At baseline assessment (Tb) all participants stated to have Internet access at home and 70.0% had a personal Facebook^®^ account for at least 3 years. The device mostly used to access their Facebook^®^ account was the smartphone (67.1%), and 55.7% of the participants reported using Facebook^®^ at least once a day.

APOLO-Teens usability was evaluated by the APOLO-Teens Usability Questionnaire (Table 1). On average, participants (N = 35) rated the intervention as highly (*n* = 15; 42.9%) to extremely useful (*n* = 8; 22.9%) and a highly (*n* = 13; 37.1%) to extremely (*n* = 9; 25.7%) useful supplement to their treatment as usual. APOLO-Teens web application (used as the self-monitoring tool) was rated as highly (*n* = 18; 51.4%)/extremely (*n* = 15; 42.9%) easy to use. Participants understood the language used in videos and images on Facebook^®^ private groups (High: *n* = 12; 34.3%/Extremely: *n* = 17; 48.6%) and found the intervention to have a significant impact on their motivation to follow medical recommendations (High: *n* = 12; 34.3%/Extremely: *n* = 8; 22.9%), highly recommending APOLO-Teens to other adolescents in the same situation (High: *n* = 15; 42.9%/Extremely: *n* = 13; 37.1%).

Over the 6-month APOLO-Teens web-based intervention, 55.26% (*n* = 21) of participants saw at least 12 out of the 24 weekly Facebook^®^ psychoeducational videos (M = 12.50, SD = 7.51) and answered to a mean of 7.68 (SD = 6.23) monitoring weekly questionnaires in APOLO-Teens web application out of 24. Regarding the monthly chat sessions, nine participants (23.7%) requested at least one chat session. These participants requested on average 1.67 (SD = 0.71) chat sessions out of 6 (min.= 1; max. = 3).

### 3.3. Intervention Effectiveness

#### 3.3.1. Primary Outcomes—Food/Beverages Consumption, Physical Activity, and Sedentary Time

To assess if patterns of change are different between the TAU group and APOLO-Teens group on behavioral outcomes, two-way mixed ANOVAs were conducted considering two-assessment moments: baseline (Tb) and end of intervention (Tf) (Table 3). The following behavioral outcome measures were evaluated: weekly consumption of soup, fruit, vegetables on the plate, sweetened beverages and pastries/cakes, minutes of sedentary time a day, minutes in moderate to vigorous physical activity at school and out of school.

Regarding the frequency of food and beverages consumption, an interaction effect between group condition and time was found (F (1,35) = 6.99, *p* = 0.012), showing different patterns of change regarding the weekly consumption of fruit between the TAU group and APOLO-Teens group. This intervention produced a large effect on fruit consumption (η_p_^2^ = 0.166). The APOLO-Teens group showed an increase in fruit consumption between baseline and end of intervention when compared with the TAU group, who decreased fruit consumption. On average, the APOLO-Teens group increased fruit consumption from five to six times per week to consume about two pieces of fruit per day. At baseline, the estimated marginal mean of the fruit consumption was 3.84 (95% CI: 2.82–4.45) in the control group and 3.39 (95% CI: 2.25–3.84) in the intervention group. At the end of the intervention, the mean in the control group remained relatively stable at 3.52 (95% CI: 2.45–4.37), whereas the intervention group showed an increase to 4.89 (95% CI: 3.54–5.41).

No significant interaction effects were found between group condition and time for weekly consumption of vegetables, soup, sweetened beverages, and pastries/cakes. However, a main effect of time was found for weekly consumption of vegetables (F (1,35) = 6.49, *p* = 0.015; η_p_^2^ = 0.157 (large effect)) and pastries/cakes (F (1,33) = 9.83, *p* = 0.004; η_p_^2^ = 0.230 (large effect)), with both groups increasing their consumption of vegetables on the plate and decreasing their consumption of pastries and cakes to less than once a week.

Two-way mixed ANOVAs (Table 3) showed a main effect of time for minutes in moderate to vigorous physical activity at school (F (1,41) = 6.62, *p* = 0.014; η_p_^2^ = 0.139 (medium effect)) and minutes on sedentary time a day (F (1,41) = 4.53, *p* = 0.039; η_p_^2^ = 0.099 (medium effect)). No interaction effects of group condition and time were found for time in sedentary activities per day and minutes in moderate to vigorous physical activity, suggesting there were no differences between the TAU and APOLO-Teens group in physical activity levels and sedentary time.

#### 3.3.2. Secondary Outcomes—Problematic Eating Behavior, Psychological Functioning, and BMI z-Score

No main effects of group condition or interaction effects were found for psychological functioning outcomes (depressive symptomatology, health-related quality of life) and problematic eating behavior (disturbed eating behavior, grazing eating pattern) (Table 4). Main effects of time were found for depressive symptomatology (F (1,71) = 4.29, *p* = 0.042; η_p_^2^ = 0.057 (small effect)) and grazing eating pattern (F (1,70) = 11.28, *p* = 0.001; η_p_^2^ = 0.139 (medium effect)), suggesting that depressive symptomatology and grazing eating type decreased in both groups between baseline and end of intervention.

Patterns of change in BMI z-score across APOLO-Teens intervention were analyzed considering anthropometric data in clinical charts for baseline and end of intervention (Table 4). There was a significant effect of time on BMI z-score (F (1,62) = 8.14, *p* = 0.006; η_p_^2^ = 0.116 (medium effect)), with BMI z-score decreasing in both groups. No interaction effect between group condition and time was found.

## 4. Discussion

To our knowledge, this is the first randomized controlled trial to demonstrate the feasibility and usability of a web-based intervention using Facebook to optimize the treatment as usual for adolescents with overweight/obesity undertreatment in public health care centers. Despite the expected high attrition in e-health trials [37,38], 87.1% of the participants completed the web-based intervention, reporting high usability and satisfaction with the several APOLO-Teens components (Facebook^®^ private group/weekly tasks, self-monitoring system with automatic feedback messages, and chat).

The standard treatment offered at low cost in public health care centers for pediatric obesity can be the first step to reducing the gap in specialized treatment access between high and low socioeconomic status. The addition of an attractive web-based CBT-based intervention to standard treatment can result, to some extent, in more favorable outcomes with low costs. Effectiveness analysis showed that the APOLO-Teens web-based intervention was superior to the standard treatment for promoting fruit consumption (group-by-time interaction effect). Over time, both groups increased their vegetable intake and decreased their consumption of pastries and cakes, time spent in moderate to vigorous physical activity at school, depressive symptoms, grazing eating patterns, and BMI z-scores. However, no significant group-by-time interaction effects were observed.

The APOLO-Teens web-based intervention was able to significantly increase fruit consumption from five to six times per week to about two portions (pieces) of fruit per day. On the other hand, the control group, receiving only the TAU, experienced a small reduction in the consumption of fruit. Even though none of the groups met the nutritional recommendations for fruits and vegetables at the end of the intervention, our data suggest that the APOLO-Teens group was closer to meeting these guidelines (which include consuming at least five portions or 400 g a day). Considering that about 78% of Portuguese adolescents do not meet this recommendation [39] these findings highlight the potential role of this online tool in promoting healthy eating.

Improving vegetable consumption may not only depend on adolescents [40]. Eating preferences and choices of household members, particularly of mothers as the usual food providers, may have a negative effect on the adolescent’s food and beverages consumption and, consequently, on their readiness and aptitude for changing eating habits [40]. Thus, increasing vegetables on the plate and soup consumption can be more challenging for adolescents with no parental support since they are frequently fully dependent on their parents to buy and prepare these healthy options. Therefore, achieving a positive change in fruit consumption may be easier than in vegetable consumption since they do not depend directly on the parents to prepare the food, but rather on fruit availability and self-motivation [41,42,43]. A decrease in pastries/cakes consumption was found for both TAU and APOLO-Teens groups. These positive results in the TAU group were expected since both groups were receiving some kind of nutritional intervention and the ingestion of high caloric foods was already low at the beginning of the intervention.

Similarly to previous studies [20], this web-based intervention was not significantly effective in increasing physical activity in adolescents with overweight and obesity. Physical activity and exercise interventions have shown small and heterogeneous effects on this population [20]. A possible explanation for these poor outcomes may include the current physical activity recommendations for youth. Recommendations suggest that children/adolescents must engage in more than 60 min of moderate to vigorous physical activity (MVPA) per day to maintain a healthy lifestyle [44], but there are no specific physical activity recommendations for weight loss in this age range. For adults, the suggested dose of physical activity to lose weight is considerably higher (~5 h/week) when compared to the recommended physical activity for health [44]. In fact, in our sample, both groups met physical activity guidelines of 60 min of moderate to vigorous physical activity (MVPA) per day at the beginning of the intervention, which may hinder further changes.

The BMI z-score decreased in both groups and there were no significant differences between the groups. Still, these results should be carefully interpreted due to the sample size and mixed literature regarding the potential effect of web-based interventions on BMI z-score [15,16,20,45]. For instance, low adherence to intervention strategies can help to explain the low effectiveness that some studies found for interventions with adolescents with overweight and obesity [46,47] since high adherence to web-based interventions has been associated with superior outcomes [48,49].

The strengths of this study include the access to the clinical population undergoing treatment in public health care, the randomized clinical trial design, the use of anthropometric data collected from clinical charts, and the manualized intervention that offered the same intervention to all the APOLO-Teens group participants. Despite the strengths, some limitations should be considered in interpreting the present study results. Primarily, the sample size, the impossibility of estimating with precision the participant’s fruit and vegetable intake per day, and data missing in some of the outcome measures precluded the use of more complex statistical models. A per-protocol analysis was chosen to assess the effects of the APOLO-Teens intervention under conditions of adequate adherence, in line with our primary objective. While we also considered conducting an intention-to-treat (ITT) analysis to account for all participants initially allocated to the intervention, this was not feasible due to the limited sample size and substantial missing outcome data. A comparison between completers and dropouts was not conducted, as it was not possible to determine with precision when or if participants disengaged from the intervention. All participants had continuous access to the Facebook-based content throughout the intervention period, and some continued to interact with the materials despite not completing the follow-up assessments.

Additionally, several variables were assessed using self-report measures (Rep(eat)-Q and YAP) and the results may be influenced by social desirability bias. Also, the use of the ChEAT total score can obfuscate potential data regarding particular disordered eating behaviors profiles.

Although overall feasibility was rated positively, some specific items received comparatively lower scores, namely, participants’ comfort in interacting with the group (3.3/5) and the perceived usefulness of the chat (3.2/5). Possible explanations include low familiarity with the platform, limited group cohesion, or a preference for more private or passive forms of interaction. To enhance engagement in future digital interventions, alternative platforms more aligned with adolescents’ communication preferences (e.g., Instagram, or WhatsApp) could be explored. Additionally, strategies such as moderated discussions, peer ambassadors, or structured interactive challenges may help foster a more dynamic and supportive online environment.

Given that the study was conducted within the Portuguese public health system—characterized by universal coverage, centralized governance, and predominantly public service provision—the generalizability of findings to other countries may be limited. Health systems with different organizational models (e.g., privatized) may encounter distinct implementation dynamics. Moreover, cultural factors such as societal attitudes toward public health interventions, trust in health care providers, and health-seeking behaviors can vary widely across countries and may influence the feasibility and effectiveness of similar interventions in other contexts. Nonetheless, the core principles underpinning the intervention, such as promoting patient engagement, targeting behavior change, and integrating multidisciplinary care are broadly applicable across diverse health care settings, particularly those committed to evidence-based practice and chronic disease prevention.

## 5. Conclusion

The results of this study reinforce the feasibility and potential of this intervention format to promote healthy eating behaviors, complementing existing adolescent weight loss practices in public health systems. Future research should include an untreated control group and a group with access solely to the web-based intervention in order to disentangle the effects of standard care and Facebook-based components. Exploring the effectiveness and adaptability of similar interventions delivered through other social media platforms will be relevant to determine whether comparable outcomes can be achieved beyond Facebook, since current trends indicate a decline in its popularity among adolescents, who increasingly favor platforms such as Instagram, TikTok, or messaging apps. To enhance engagement and reach, future adaptations should consider more widely used platforms among the target population, ensuring alignment with their digital habits and preferences.

## Figures and Tables

**Table 1:** APOLO-Teens feasibility and usability.

Items	Mean (SD)
Apolo-Teens Facebook^®^ Private Group	
I had problems accessing/finding the APOLO-Teens group on Facebook.	0.26 (0.51)
I was able to see/find the videos and images available in the Facebook group of APOLO-Teens.	2.69 (1.51)
The videos and images posted on the APOLO-Teens Facebook group every week were helpful.	2.43 (1.12)
The language used in the videos and images published in the APOLO-Teens Facebook group was easy to understand.	3.23 (0.97)
The videos and images posted in the Facebook group were important to motivate me to change my behaviors.	2.49 (1.15)
I felt comfortable interacting with the group and making comments and giving likes on the videos/images available in the Facebook group of APOLO-Teens.	1.89 (1.43)
I liked having access to the APOLO-Teens Facebook group.	3.11 (0.96)
Weekly Tasks	
I had difficulty understanding video information and posts about weekly assignments.	57 (0.98)
The tasks I had to do each week were easy to accomplish.	2.29 (1.02)
Weekly tasks were helpful to me.	2.60 (0.98)
I enjoyed doing the weekly tasks.	2.37 (1.00)
Weekly self-monitoring system with automatic feedback messages	
I like the visual aspect of the APOLO-Teens website.	2.86 (1.06)
The APOLO-Teens website is easy to use.	3.29 (0.86)
I had difficulty accessing/answering the weekly questionnaire on the APOLO-Teens website.	0.60 (0.85)
The Sunday message to remember to respond to the weekly questionnaire was useful.	3.03 (1.29)
The tips given in the messages that I received at the end of the weekly questionnaire helped improve my eating and physical activity habits.	2.43 (1.07)
I find the weekly questionnaire useful.	2.80 (1.02)
I liked responding to the weekly questionnaire.	2.31 (1.13)
Chat sessions	
I had difficulty using the “Chat” feature on Facebook.	0.19 (.51)
In the chat sessions, the professional helped me to overcome my problems and clarify my doubts.	1.95 (1.58)
I think that the duration of the chat session was sufficient.	1.58 (1.50)
I liked the service provided by the professional in the chat.	1.95 (1.62)
Chat sessions were useful.	2.22 (1.63)
Perception of satisfaction/Overall Utility	
The APOLO-Teens program is a useful complement to the consultations that I have in the hospital.	2.66 (1.19)
The APOLO-Teens program helps me to stay motivated and comply with my doctor’s recommendations.	2.54 (1.17)
Throughout the program, I have changed some of the less healthy behaviors I had.	2.20 (1.08)
I am satisfied with the results I am getting, with the help of the APOLO-Teens program.	2.20 (1.13)
In general, I am satisfied with the APOLO-Teens intervention program	2.80 (0.96)
In general, I think this program is useful to me.	2.66 (1.11)
I would recommend this program to other teens who have overweight or obesity.	3.06 (1.00)

Note: *n* = 35; Likert scale: 0 = Nothing; 1 = Slightly; 2 = Moderately; 3 = Highly; 4 = Extremely.

**Table 2:** Baseline characteristics of participants in the APOLO-Teens randomized controlled trial.

	TAU Control Group (*n* = 39)	APOLO-Teens Group (*n* = 38)	* p*-Value
Adolescents			
Age, years	14.87 (1.58)	14.97 (1.68)	0.81 ^a^
Sex			0.26 ^b^
Female, *n* (%)	24 (61.50)	28 (73.7)	
Male, *n* (%)	15 (38.50)	10 (26.30)	
BMI kg/m^2^, mean (SD)	29.50 (4.03)	29.61 (4.72)	0.92 ^c^
BMI, z-score, mean (SD)	2.29 (0.64)	2.30 (0.69)	0.83 ^c^
BMI Status–WHO			0.75 ^b^
Overweight, *n* (%)	13 (33.30)	14 (36.80)	
Obesity, *n* (%)	26 (66.70)	24 (63.20)	
Parents and Household			
Mother age, mean (SD), y	43.51 (5.52)	45.19 (4.56)	0.15 ^c^
Father age, mean (SD), y	47.13 (6.35)	47.31 (5.39)	0.90 ^c^
Mother educational attainment			0.59 ^b^
≤Elementary school	7 (17.95)	3 (7.89)	
≤High school degree	28 (71.79)	30 (78.95)	
≥Bachelors degree	4 (10.26)	5 (13.16)	
Father educational attainment *			0.20 ^b^
≤Elementary school	6 (15.79)	4 (11.11)	
≤High school degree	30 (78.95)	27 (75.00)	
≥Bachelors degree	2 (5.26)	5 (13.89)	
Number of siblings, mean (SD)	1.16 (1.41)	1.06 (0.74)	0.74 ^a^

Note: N = 77; TAU control group = treatment as usual control group; APOLO-Teens Group = treatment as usual control group plus APOLO-Teens web-based intervention; BMI= Body Mass Index; BMI Status–WHO = WHO BMI z-score cutoffs: overweight = > 1; obesity = > 2; ^a^ Mann–Whitney Test; ^b^ Chi-square Test (χ2); ^c^ *t* test for independent samples; * there are missing date.

**Table 3:** Summary of two-way ANOVA results for primary outcomes.

.	TAU Control Group (*n* = 39)	APOLO-Teens Intervention Group (*n* = 38)	Two-Way ANOVA				
	*M (SD)*	*M (SD)*	MS	*df*	*F*	*p*	Effect Size (η_p_^2^)
Behavioral Outcomes							
*Fruit Consumption (weekly intake)*				(1,35)			
Baseline (Tb)	3.84 (1.86)	3.39 (1.65)					
End of intervention (Tf)	3.52 (2.17)	4.89 (2.17)					
Time			6.48		2.97	0.093	0.078
Group Condition			3.82		0.679	0.416	0.019
Time × Group Condition			15.24		6.99	0.012 *^a^	0.166
*Vegetables on the plate Consumption (weekly intake)*				(1,35)			
Baseline (Tb)	3.16 (2.67)	2.61 (2.30)					
End of intervention (Tf)	3.37 (1.53)	4.00 (1.91)					
Time			11.82		6.49	0.015 *	0.157
Group Condition			0.033		0.004	0.947	0.000
Time × Group Condition			6.42		3.53	0.069	0.091
*Soup Consumption (weekly intake)*				(1,35)			
Baseline (Tb)	2.89 (2.13)	3.22 (1.73)					
End of intervention (Tf)	3.53 (2.37)	3.56 (1.82)					
Time			4.30		1.69	0.202	0.046
Group Condition			0.588		0.102	0.751	0.003
Time × Group Condition			0.411		0.161	0.690	0.005
*Sweetened beverages Consumption (weekly intake)*				(1,35)			
Baseline (Tb)	1.37 (1.34)	1.67 (1.57)					
End of intervention (Tf)	1.21 (0.98)	0.94 (1.16)					
Time			3.58		2.55	0.119	0.068
Group Condition			0.005		0.003	0.960	0.000
Time × Group Condition			1.47		1.05	0.313	0.029
*Pastries/sweets Consumption (weekly intake)*				(1,33)			
Baseline (Tb)	1.22 (1.26)	1.18 (1.19)					
End of intervention (Tf)	0.50 (0.51)	0.76 (0.75)					
Time			5.62		9.83	0.004 **	0.230
Group Condition			0.210		0.156	0.695	0.005
Time × Group Condition			0.421		0.737	0.397	0.022
*Minutes in moderate to vigorous physical activity at school (YAP)*				(1,41)			
Baseline (Tb)	54.89 (11.50)	52.47 (15.37)					
End of intervention (Tf)	52.00 (14.33)	50.05 (16.08)					
Time			151.34		6.62	0.014 *	0.139
Group Condition			102.65		0.260	0.613	0.006
Time × Group Condition			1.18		0.051	0.822	0.001
*Minutes in moderate to vigorous physical activity out of school (YAP)*				(1,41)			
Baseline (Tb)	58.12 (12.12)	58.84 (14.61)					
End of intervention (Tf)	57.07 (10.95)	57.34 (14.82)					
Time			34.93		1.17	0.286	0.028
Group Condition			5.29		0.016	0.899	0.000
Time × Group Condition			1.07		0.036	0.851	0.001
*Minutes of sedentary time a day (YAP)*				(1,41)			
Baseline (Tb)	288.98 (26.38)	300.92 (37.87)					
End of intervention (Tf)	305.96 (36.09)	301.64 (33.68)					
Time			1681.86		4.53	0.039 *	0.099
Group Condition			312.30		0.163	0.689	0.004
Time × Group Condition			1418.58		3.82	0.058	0.085

Note: M—mean; SD—standard deviation; MS— mean squares; YAP—Youth Activity Profile; η_p_^2^—Partial Eta Squared; * *p* < 0 .05, ** *p* <  0.01. ^a^ Time × Group Condition significant interaction effect.

**Table 4:** Summary of two-way ANOVA results for secondary outcomes.

.	TAU Control Group (*n* = 39)	APOLO-Teens Intervention Group (*n* = 38)	Two-Way ANOVA				
	*M (SD)*	*M (SD)*	MS	*df*	*F*	*p*	Effect Size (η_p_^2^)
Problematic eating behavior							
*Disturbed eating behavior (ChEAT total score)*				(1,70)			
Baseline (Tb)	16.31 (8.40)	18.97 (10.80)					
End of intervention (Tf)	15.14 (7.43)	18.38 (9.25)					
Time			28.05		1.09	0.300	0.015
Group Condition			312.43		2.24	0.139	0.031
Time × Group Condition			2.99		0.116	0.734	0.002
*Grazing eating pattern (Rep(eat)-Q total score*				(1,70)			
Baseline (Tb)	1.84 (1.62)	1.47 (1.34)					
End of intervention (Tf)	1.28 (1.24)	1.06 (1.23)					
Time			8.49		11.28	0.001 **	0.139
Group Condition			3.10		1.04	0.311	0.015
Time × Group Condition			.208		0.276	0.601	0.004
Psychological functioning							
*Depressive symptomatology (DASS-21)*				(1,71)			
Baseline (Tb)	5.72 (5.74)	4.97 (4.63)					
End of intervention (Tf)	3.94 (5.77)	4.24 (4.96)					
Time			57.36		4.29	0.042 *	0.057
Group Condition			1.85		0.043	0.836	0.001
Time × Group Condition			10.02		0.749	0.390	0.010
*Health-related quality of life (PedsQL* ^TM^ *total score)*				(1,70)			
Baseline (Tb)	79.44 (16.03)	74.25 (15.43)					
End of intervention (Tf)	79.32 (15.21)	76.27 (17.95)					
Time			32.56		0.663	0.418	0.009
Group Condition			611.49		1.29	0.260	0.018
Time × Group Condition			41.36		0.842	0.362	0.012
BMI z-score §				(1,62)			
Baseline (Tb)	2.29 (0.64)	2.30 (0.69)					
End of intervention (Tf)	2.25 (0.57)	2.17 (0.70)					
Time			0.226		8.14	0.006 **	0.116
Group Condition			0.043		0.053	0.818	0.001
Time × Group Condition			0.060		2.16	0.147	0.034

Note: M—mean; SD—standard deviation; MS—mean squares; ChEAT—Children’s Eating Attitudes Test; Rep(eat)-Q—Repetitive Eating Questionnaire; DASS-21—Depression Anxiety Stress Scales; PedsQL^TM^—Pediatric Quality of Life Inventory; η_p_^2^—Partial Eta Squared; § BMI z-score from clinical charts; * *p*  <  0.05, ** *p*  <  0.01.

## Data Availability

The datasets generated during and/or analyzed during the current study are available from the corresponding author on reasonable request.

## Supplementary Materials

The following supporting information can be downloaded at https://www.mdpi.com/article/10.3390/nu17162586/s1; Figure S1: APOLO-Teens Facebook Intervention Interface; Figure S2: APOLO-Teens Web-Aplication Intervention Interface.

## Abbreviations

The following abbreviations are used in this manuscript:

BMI	Body Mass Index
MVPA	Moderate-to-vigorous physical activity
TAU	Treatment As Usual
Tb	Treatment Basline Assessment
Tf	End of intervention Assessment
