# Supplementary material for: Feasibility and Effectiveness of a Social Network-Based Intervention for Adolescents Undergoing Weight Loss Treatment: A Randomized Controlled Trial
Source: Nutrients. 2025 Aug 8;17(16):2586. doi: 10.3390/nu17162586 (PMC12389724; doi:10.3390/nu17162586)

Feasibility and Effectiveness of a Facebook-based Intervention for Adolescents Undergoing Weight Loss Treatment: A Randomized Controlled Trial

Figure S1. APOLO-Teens Facebook Intervention Interface

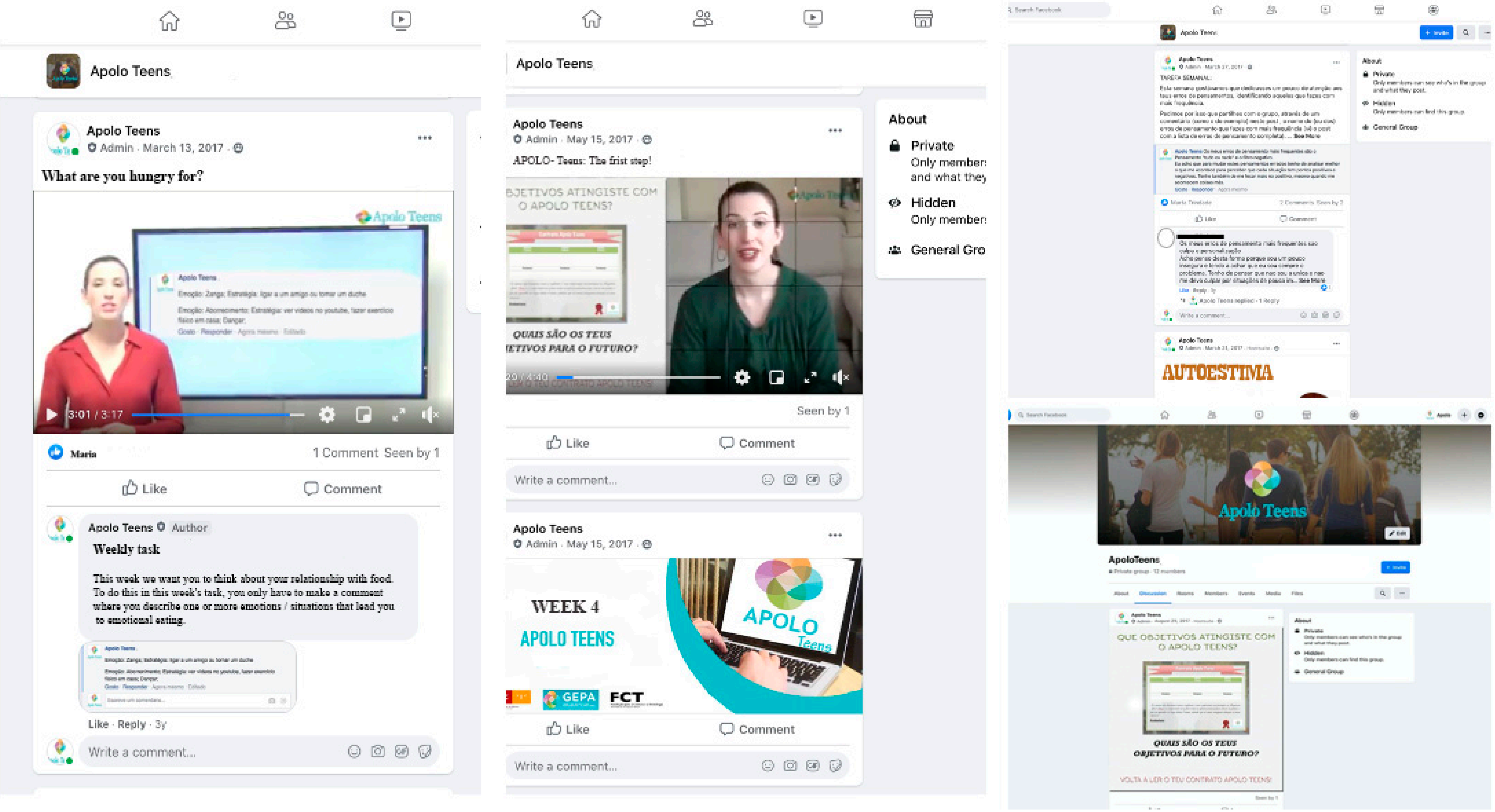

Figure S2. APOLO-Teens Web-Aplication Intervention Interface

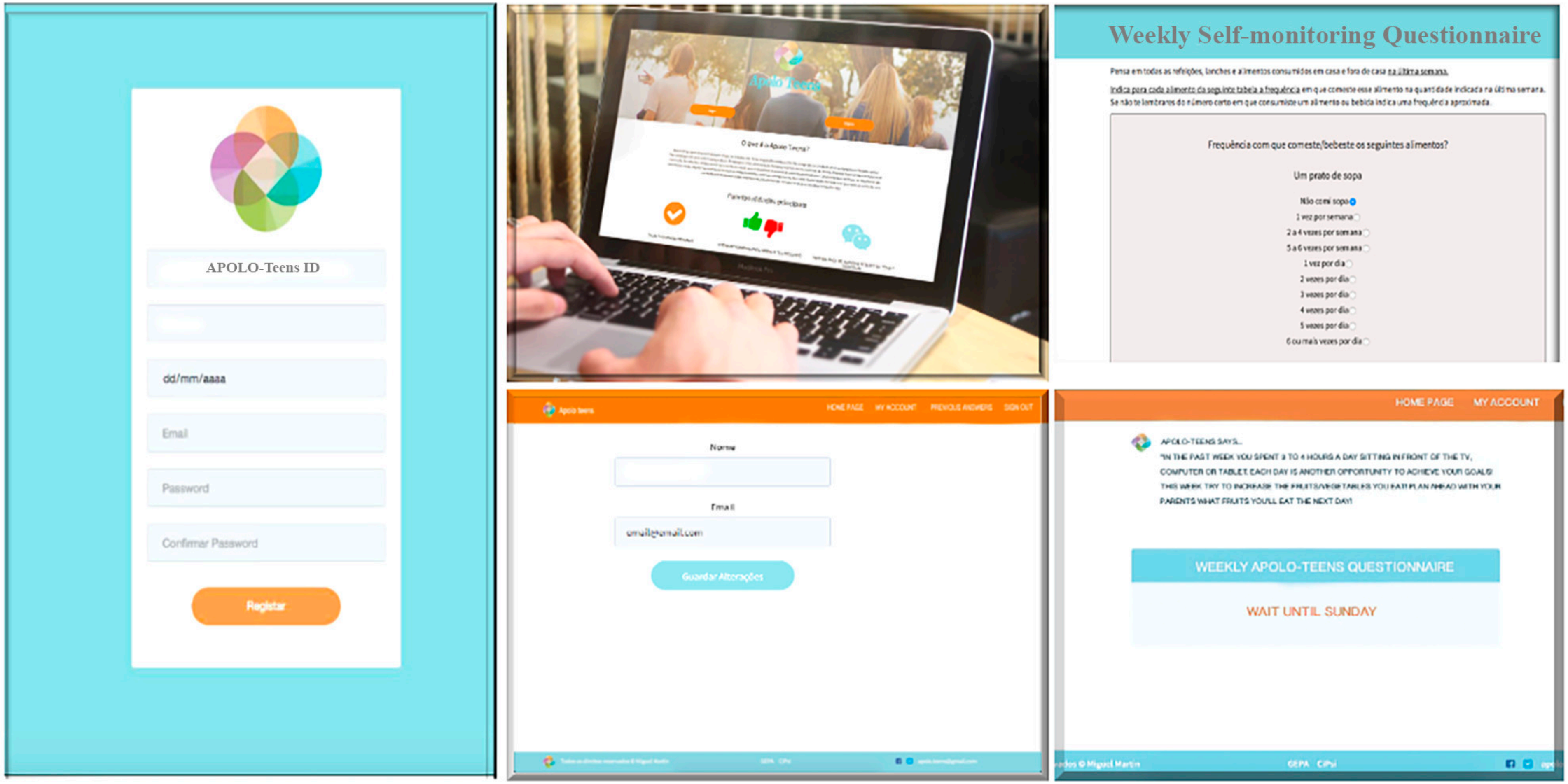

Supplement: Supplementary file 1 [file nutrients-17-02586-s001.zip › nutrients-3790152-supplementary.pdf]
